# Limited spatiotemporal niche partitioning among mesocarnivores in Gorongosa National Park, Mozambique

**DOI:** 10.1002/ece3.10965

**Published:** 2024-02-15

**Authors:** Kathryn L. Grabowski, Erin M. Phillips, Kaitlyn M. Gaynor

**Affiliations:** ^1^ School of Geography and the Environment University of Oxford Oxford UK; ^2^ Department of Biology McGill University Montréal Québec Canada; ^3^ Department of Ecology and Evolutionary Biology Princeton University Princeton New Jersey USA; ^4^ Department of Zoology and Botany University of British Columbia Vancouver British Columbia Canada

**Keywords:** camera trap, competition, mesopredator, occupancy, restoration, Southern Africa

## Abstract

Competition drives community composition and structure in many ecosystems. Spatial and temporal niche partitioning, in which competing species divide the environment in space or time, are mechanisms that may allow for coexistence among ecologically similar species. Such division of resources may be especially important for carnivores in African savannas, which support diverse carnivore assemblages. We used camera traps to explore patterns of spatial and temporal niche partitioning among four mesocarnivore species in Mozambique's Gorongosa National Park: large‐spotted genet (*Genetta maculata*), African civet (*Civettictis civetta*), honey badger (*Mellivora capensis*) and marsh mongoose (*Atilax paludinosus*). We applied a multispecies occupancy model to evaluate spatial partitioning among mesocarnivores and to quantify the environmental factors that affect species‐specific habitat use, including relative lion (*Panthera leo*) activity. We also analyzed the temporal activity overlap of the four focal species. We identified species‐specific habitat covariates that influenced detection probabilities but found no evidence of spatial or temporal partitioning among mesocarnivores in the study system. Indeed, we found some evidence for spatial co‐occurrence between two of our focal species: African civet and marsh mongoose. There may be limited competition among mesocarnivores in this system, perhaps due to niche and diet differentiation among these species and an abundance of resources. While we found limited evidence that lion activity impacts mesocarnivores, ongoing monitoring of intraguild interactions is vital as apex predator populations recover in the system. This study adds to a growing understanding of African mesocarnivore ecology and highlights the importance of understanding these dynamics for effective multispecies conservation and restoration.

## Graphical abstract

We used camera traps to explore patterns of spatial and temporal niche partitioning as mechanisms supporting coexistence among four mesocarnivore species in Mozambique's Gorongosa National Park. We found no evidence of spatial or temporal avoidance among species, and we documented species‐specific environmental factors that affected detection probability. This study contributes valuable insights into African mesocarnivore ecology, particularly in the absence of apex predators, and highlights the importance of understanding these dynamics for effective conservation and restoration.

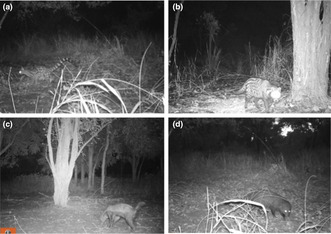

## 1 INTRODUCTION

By the competitive exclusion principle, species that are too ecologically similar cannot coexist, as one species will eventually outcompete another (Gause, 1934; Levin, 1970). As such, competition is an important force driving community structure. Mammalian carnivores exhibit high degrees of sympatry, similarities in features like body size and diet, and potential for intraguild predation, all of which contribute to the role of competition as an important driver of intraguild interactions in carnivores (Donadio & Buskirk, 2006; Fedriani et al., 2000; Holt & Huxel, 2007). Competition among carnivores may take different forms, including exploitation competition, an indirect interaction in which species use the same resources, and interference competition, in which one species directly affects the ability of other species to access resources (Bianchi et al., 2016; Birch, 1957; Gompper et al., 2016). Such competitive interactions often have important impacts on the realized niches of carnivorous mammals (Creel & Creel, 1996; Fedriani et al., 1999; Linnell & Strand, 2000).

Niche partitioning, in which competing species use the environment differently, can reduce the impacts of competition on carnivore populations and thus allow for the coexistence of multiple carnivore species (Di Bitetti et al., 2009; Kamler et al., 2012; Linnell & Strand, 2000). Within carnivore guilds, less dominant competitors may alter their habitat use or behavior, including foraging patterns and activity budgets, in the presence of dominant competitors (de Satgé et al., 2017; Johnson & VanDerWal, 2009; Sergio & Hiraldo, 2008). Spatiotemporal niche partitioning has been documented in many systems (e.g., Bianchi et al., 2016; Di Bitetti et al., 2009; Palomares et al., 1996) as a way to minimize the risk of negative competitive interactions. For example, cheetahs (*Acinonyx jubatus*), African wild dogs (*Lycaon pictus*), and leopards (*Panthera pardus*) often avoid resource‐rich habitats and alter their activity timing in the presence of lions (*Panthera leo*), which are larger and more dominant competitors (Durant, 1998; Swanson et al., 2014; Vanak et al., 2013). Ecologically similar species may also differentiate their habitat use to reduce competition, either over evolutionary time scales or as a plastic phenotypic response to the presence of competitors (Comley et al., 2020a, 2020b; Shen et al., 2021). Dietary niche partitioning also facilitates coexistence, reducing both interference and exploitation competition over shared prey species (Carvalho & Gomes, 2004; Lu et al., 2023; Owen‐Smith & Mills, 2008).

While there is strong evidence of spatiotemporal partitioning among larger carnivores, less is known about the competitive interactions of mesocarnivores. Mesocarnivores, which are smaller‐bodied carnivores occupying intermediate trophic positions (Prugh et al., 2009), have historically been difficult to study due to their rare, elusive, and nocturnal nature. Motion‐activated camera traps, however, generate data on animal activity without the need for a human observer and are therefore ideally suited for documenting the spatiotemporal niches of mesocarnivores (Frey et al., 2017; Rowcliffe et al., 2008; Silveira et al., 2003). Many studies have used camera traps to assess mesocarnivore activity, with some finding evidence of both spatial and temporal partitioning (Easter et al., 2020; Mills et al., 2019; Tsunoda et al., 2020), and others finding evidence of spatial co‐occurrence among ecologically similar mesocarnivores (Monterroso et al., 2020; Pereira et al., 2012). Despite these insights, studies of mesocarnivore niche partitioning have historically been restricted to North America and Europe, with more recent emerging knowledge of African systems (Shores et al., 2019; Tsunoda et al., 2020; Welch et al., 2023; Easter et al., 2020; see also Bianchi et al., 2016; Watabe et al., 2022 for South America, Asia).

African savannas are home to some of the planet's most diverse carnivore guilds, with as many as 20 carnivore species co‐occurring in a single ecosystem (Do Linh San et al., 2013; Schuette et al., 2013). Overlapping geographic ranges, habitats, and diets result in high potential for interspecific competition among these species, thus presenting ideal environments in which to study the ecological and behavioral mechanisms that facilitate carnivore coexistence (Caro & Stoner, 2003; Rich et al., 2017). For example, recent work suggests that unique combinations of variables might affect the spatial dynamics of different carnivore species in an African system, with smaller carnivore detections influenced mostly by large carnivores and large carnivore detections mostly influenced by environmental factors (Comley et al., 2020b). Additionally, many African mesocarnivore species are similar in size, which strongly impacts the likelihood of competition due to the use of overlapping resources (Donadio & Buskirk, 2006; Hutchinson & MacArthur, 1959; Ritchie & Johnson, 2009). Previous research has documented dietary overlap among African mesocarnivores, including between large‐spotted genets (*Genetta maculata*, hereafter “genet”) and African civets (*Civetticis civetta*, hereafter “civet”), and between genets and marsh mongooses (*Atilax paludinosis*) (Angelici, 2000), and spatial overlap between genets and slender mongooses (*Galerella sanguinea*) (Maddock & Perrin, 1993). While these studies have provided important insights into African mesocarnivore ecology, there remains a gap in our understanding of whether and how multiple mesocarnivore species partition space and time on fine scales to facilitate coexistence.

Here, we aim to address this gap by presenting a study of the mesocarnivore community in Gorongosa National Park (GNP) in Mozambique. The large carnivore populations in the ecosystem were nearly extirpated as a result of hunting during the Mozambican civil conflict, which provides a unique opportunity to understand niche partitioning among mesocarnivores in the near absence of predation and competition from larger predators (Gaynor et al., 2021). Previous work in this system has reported spatial avoidance between civets and genets as a potential mechanism for coexistence (Raimundo, 2020). However, another study from the greater Gorongosa ecosystem found limited evidence of spatial or temporal partitioning between civets and genets and instead found evidence of spatial and temporal avoidance between civets and bushy‐tailed mongooses (*Bdeogale crassicauda*) (Easter et al., 2020). Understanding the system's dynamics at this stage will benefit conservation efforts by increasing our knowledge of ecosystems without apex predators, which are becoming increasingly common worldwide (Estes et al., 2011; Ripple et al., 2014). This work will also provide a baseline with which to compare future states of the Gorongosa ecosystem as it undergoes active restoration of large carnivores.

In this study, we sought to assess patterns of spatial and temporal partitioning among the four most commonly detected mesocarnivore species in GNP (civets, genets, honey badgers [*Mellivora capensis*] and marsh mongooses), and to quantify their habitat associations. Specifically, we asked: (1) What is the degree of spatial and temporal niche partitioning among mesocarnivores in GNP? and (2) What environmental features are associated with the spatial distribution of each mesocarnivore species?

We expected to see spatial and/or fine‐scale temporal niche partitioning between species pairs that exhibit a strong overlap in diet (e.g., genets and civets, or genets and marsh mongooses) (Angelici, 2000) or body size (e.g., civets and honey badgers or marsh mongooses and genets) (Di Bitetti et al., 2009; Schuette et al., 2013). Conversely, mesocarnivores with similar ecological traits may have a high likelihood of co‐occurrence as they are drawn to the same features of the landscape (Davies et al., 2007; Davis et al., 2018). Mesocarnivores also exhibit high ecological generalism and flexibility, which may allow them to avoid competition (Monterroso et al., 2020; Prugh et al., 2009).

We predicted that three environmental features would be correlated with mesocarnivore space use: distance to available water (for civet, genet, and marsh mongoose), percent tree cover (for civet and genet), and termite mound density (for civet, genet, and honey badger). Civets, genets and marsh mongooses are all known to prefer habitats closer to water sources both for drinking and available prey (Kingdon, 1977; Rosevear, 1974; Skinner & Chimimba, 2005). Different mesocarnivore species have varied preferences for tree cover, with civets typically found in areas with less tree cover (Grubb et al., 1998; Kingdon & Hoffmann, 2013; Raimundo, 2020) and genets as the most arboreal of the four focal species (Maddock & Perrin, 1993). Mesocarnivore species, especially honey badgers, often rely on termite mounds for cover during their rest periods or as a place to find food (Kingdon & Hoffmann, 2013), as they are important sources of termites, fruits and small mammals (Fleming & Loveridge, 2003; Okullo et al., 2013).

We also predicted that mesocarnivore space use would be affected by the presence of a dominant apex carnivore, lions (e.g., Comley et al., 2020b; Swanson et al., 2014; Vanak et al., 2013). However, given the small population of lions present in the park during the study period (Bouley et al., 2018) and the lack of ubiquitous top‐down effects in complex African systems (Comley et al., 2020b), we alternatively predicted that mesocarnivore species would not be affected by lion activity (Brodie et al., 2018).

## 2 METHODS

### 2.1 Study site

Gorongosa National Park (GNP) is in central Mozambique at the southern extent of the Great Rift Valley. The protected area encompasses a variety of landscapes, including grassland, savanna, and woodland habitats, as well as seasonally inundated floodplain that surrounds Lake Urema (Herrero et al., 2020; Stalmans & Beilfuss, 2008). The 4000 km^2^ park experiences distinct wet and dry seasons, with most of the 700–900 mm of annual rainfall in the park's interior during the wet season from December to March.

Mozambique experienced a civil conflict from 1977 to 1992 (Pringle, 2017), which decimated the mammalian community within GNP (Cumming et al., 1994) and had an especially large impact on the park's large carnivores. While the pre‐conflict community included lions, leopards, spotted hyenas (*Crocuta crocuta*), and African wild dogs (Stalmans et al., 2019), only lions survived the conflict, with a significantly smaller population (Bouley et al., 2018). None of the other larger predators had re‐established permanent populations within the park's boundaries by the time of this study. Twelve mesocarnivore species have been detected in the park since the conflict: civets, genets, honey badgers, servals (*Leptailurus serval*), side‐striped jackals (*Canis adustus*), and seven species of mongoose (Gaynor et al., 2021; Stalmans et al., 2019).

### 2.2 Camera trap grid

**FIGURE 1:**
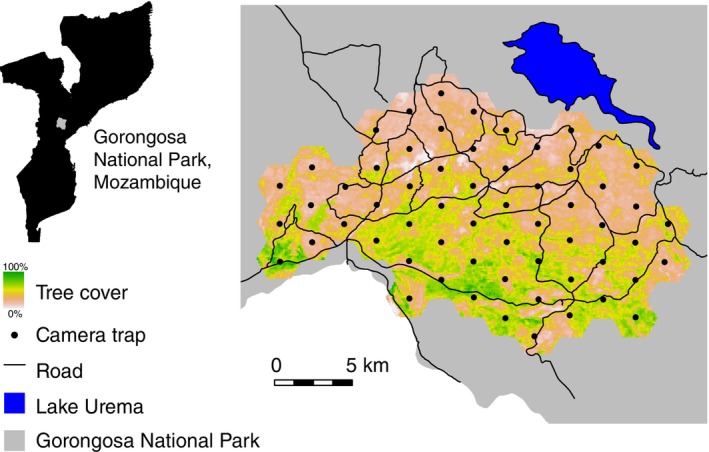
Study area in Gorongosa National Park. The camera trap grid is located south of Lake Urema, in savanna woodland. Insets show the location of Gorongosa National Park within Mozambique, and the study area within the park. Reproduced from Gaynor et al. (2021) with permission from Wiley.

We used camera traps to examine spatiotemporal patterns of mesocarnivore activity at GNP. We arranged 60 motion‐activated camera traps (Bushnell TrophyCam) in a grid in a 300 km^2^ area of woodland south of Lake Urema (Figure 1). We used data from the 2016 late dry season (as defined by Bouley et al., 2018; August–November; 122 days) to better meet model assumptions of closure (Sollmann, 2018). We chose the late dry season because there is less vegetation during that time to obscure mesocarnivores, and because resource scarcity might be associated with the strongest patterns of spatiotemporal niche partitioning. Additionally, Raimundo (2020) reported that civet and genet detection probabilities were highest in the dry season. Nine of the cameras were inoperable for some portion of the study period, but we retained them in the analysis as all were active for at least 23 days.

Details on camera placement can be found in Gaynor et al. (2021). After identifying all photographed animals to species level, we generated a detection record using the camtrapR package (Niedballa et al., 2016) in R v3.6.3 (R Core Team, 2020). We considered a species to be detected on a given date if there was at least one photograph containing the species and undetected if not.

### 2.3 Spatial analysis

**FIGURE 2:**
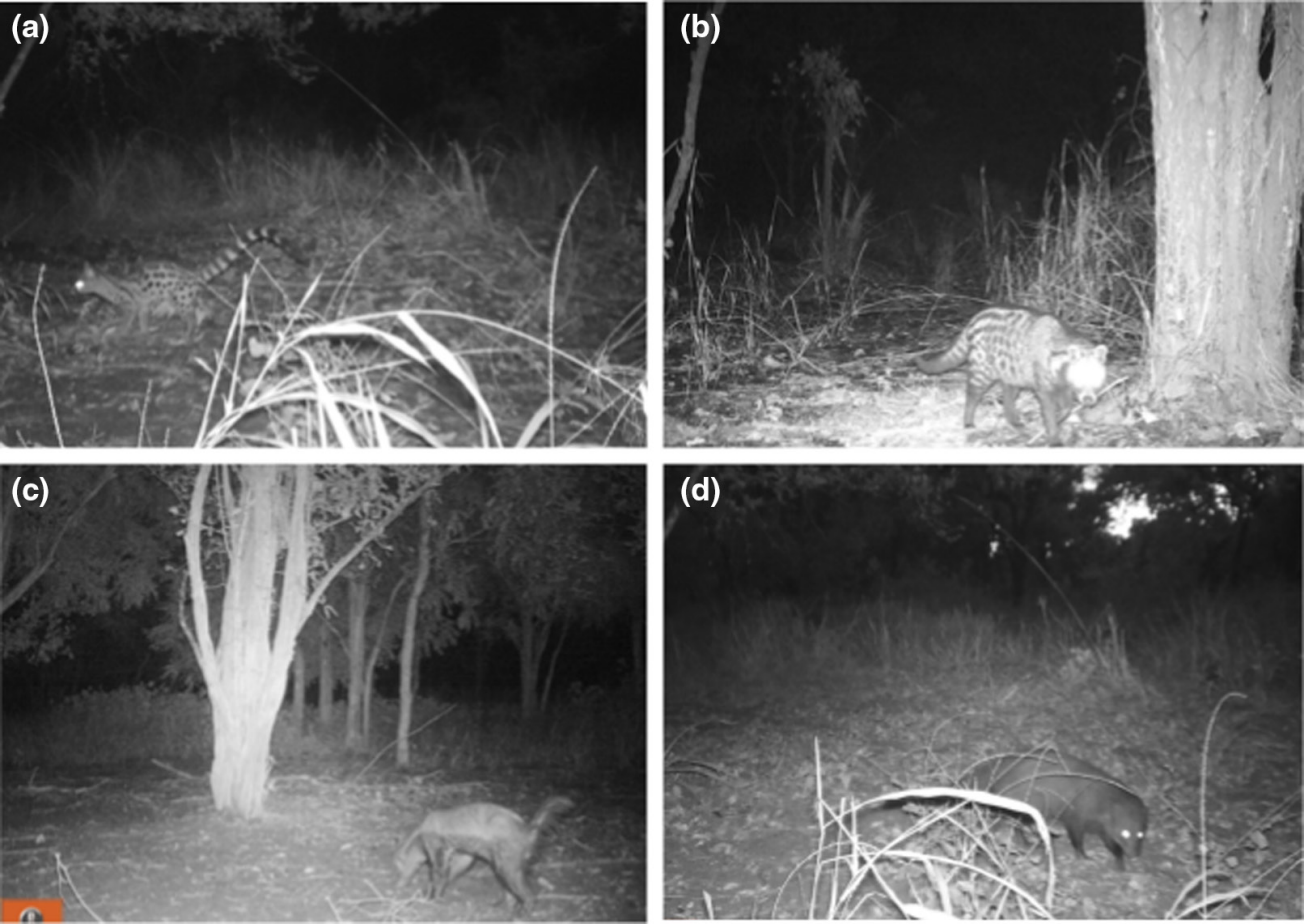
Camera trap images of the four focal species: (a) genet, (b) civet, (c) honey badger, and (d) marsh mongoose.

We applied a multispecies conditional occupancy modeling approach developed by Rota et al. (2016) to study the spatial distribution and co‐occurrence of four focal mesocarnivore species: civet, genet, honey badger, and marsh mongoose (Figure 2). We chose these species because they were the most commonly detected (at least 80 detections each during our study period) and together they have strong potential for interspecific competition based on their ecology. This modeling approach allows concurrent assessments of the latent occupancy state of multiple species relative to environmental variables and the presence or absence of other potentially interacting species (Miller et al., 2018). It also provides detection probability estimates of multiple species relative to environmental variables. This framework assumes that the latent occupancy state is drawn from a multivariate Bernoulli (MVB) distribution, which overcomes limitations associated with previous multispecies models that incorporated species interactions and required an a priori assumption about species' dominant and subordinate status (e.g., Richmond et al., 2010). We used a maximum likelihood estimation technique to fit this model.

We used the following spatial covariates in our models: termite mound density, distance to water, tree cover, and lion activity. We determined termite mound density within a 1 km radius of the camera based on a LiDAR‐generated map of termite mound distribution (Daskin et al., 2023). We chose this radius because it falls within the span of reported home range estimates for our four focal species (Admasu et al., 2004; Baker & Ray, 2013; Begg et al., 2013; Roux et al., 2016). Available water included rivers, large pans (>1 km^2^ in area, hand‐digitized from 2015 DigitalGlobe imagery), and Lake Urema (dry season extent) and was incorporated into a single raster layer. For the distance to water covariate, we used this raster layer to calculate distance from each camera to the closest available water. For tree cover, we used a tree cover raster from the Global Forest Change database, which mapped global forest loss at a spatial resolution of 30 m (Hansen et al., 2013). We extracted the layer's value at each camera site using the raster package (Hijmans, 2020) in R v3.6.3 (R Core Team, 2020).

There were insufficient lion camera trap detections (12 during the study period) to incorporate lions directly into our spatial models, so we used a proxy of lion activity generated with satellite collar data from Bouley et al. (2018). Bouley et al. (2018) produced late dry season (August–November) home‐range isopleths for 16 collared individuals from 2013, 2014, and 2015 at the 0.10, 0.25, 0.50, 0.75, and 0.95 levels. We created an index of relative activity of each lion, ranging from 0 to 5, where 0 corresponded to areas outside of the 0.95 isopleths, 1 corresponded to areas that fell only in the 0.95 isopleth (containing 95% of GPS points), 2 to the 0.75 isopleth, 3 to the 0.50 isopleth, 4 to the 0.25 isopleth, and 5 to the 0.10 isopleth (tight core of a lion's home range). When more than 1 year's set of isopleths was available for an individual lion, we used the most recent to create its raster layer to avoid replication. We then added layers from every lion together. Although we do not have lion isopleths from the same year as our camera trap data, the overall lion population size did not vary much between 2013 and 2015 (time period of GPS points) and 2016 (time period of our camera trap data) (Bouley et al., 2018), nor did the general spatial distribution and habitat associations of lions. However, we do recognize that our lion covariate is a very coarse approximation of lion activity in the study area.

We adopted a two‐stage model fitting approach (Richmond et al., 2010). In the first stage, we determined the most informative combination of occupancy and detection covariates in exploratory single‐species models to subsequently use in the multispecies models. We also tested whether two predictors of detection probability related to the camera placement improved our models: a binary measure of whether the camera's maximum 10 m range was obscured, and the percentage of ground cover in a 10 m radius around the camera (percentage of ground that was not bare soil, estimated visually by a single observer). These might influence detection rates of smaller‐bodied mammals like mesocarnivores. These detection covariates consistently improved model fit in the single‐species exploration, so we retained them in all future models.

Based on the literature and personal observations of the four focal species, we explored the following environmental covariates for each species: for genet and civet – distance to water, termite mound density, tree cover, and lion activity; for honey badger – termite mound density and lion activity; and for marsh mongoose – distance to water and lion activity. These environmental covariates may affect where a species is present, reflected in its occupancy. They may also affect species density and intensity of use of a site, and thus influence detection probabilities (Hofmeester et al., 2019). As such, we evaluated models including these covariates in both the occupancy and the detection probability formulas (Tables 1, 2, 3, 4). Prior to modeling, we scaled the values of all continuous covariates to have a mean of 0 and a standard deviation of 1. For each species, we first identified the models within 2 ΔAIC of the model with the lowest AIC (Akaike information Criterion; Akaike, 1974). Out of these top models, we selected the model with the fewest number of covariates to minimize the number of parameters in our multispecies models.

**TABLE 1:** Genet single‐species occupancy model results. Under “Covariates”, ‘O’ means that the variable was included in the model as an occupancy covariate and ‘D’ means it was included as a detection covariate. The model in bold includes the combination of covariates that we carried through to the multispecies models.

Model name	Covariates						AIC	ΔAIC	*wᵢ* (AIC)
	Range obscured	Ground cover	Termite mound density	Distance to water	Tree cover	Lion activity			
G16	D	D	D	D	D		5162.66	0	0.18
**G8**	**D**	**D**			**D**		**5162.70**	**0.04**	**0.17**
G13	D	D		D	D		5162.71	0.05	0.17
G11	D	D	D		D		5162.75	0.09	0.17
G20	D	D	D	D	D	D	5164.38	1.71	0.08
G17	D	D		D	D	D	5164.38	1.72	0.07
G15	D	D			D	D	5164.39	1.72	0.07
G18	D	D	D		D	D	5164.48	1.82	0.07
G7	D	D		D			5170.1	7.44	<0.001
G14	D	D		D		D	5170.99	8.33	<0.001
G10	D	D	D	D			5171.76	9.1	<0.001
G19	D	D	D	D		D	5172.63	9.97	<0.001
G1	D	D					5174.95	12.28	<0.001
G9	D	D				D	5175.61	12.95	<0.001
G4	D	D			O		5176.77	14.11	<0.001
G5	D	D				O	5176.78	14.12	<0.001
G3	D	D		O			5176.84	14.17	<0.001
G6	D	D	D				5176.85	14.19	<0.001
G2	D	D	O				5176.91	14.25	<0.001
G12	D	D	D			D	5177.51	14.84	<0.001
G0							5287.5	124.84	<0.001

Abbreviations: AIC, Akaike Information Criterion; 𝑤𝑖 (AIC), Akaike weight.

**TABLE 2:** Civet single‐species occupancy model results. Under “Covariates”, ‘O’ means that the variable was included in the model as an occupancy covariate and ‘D’ means it was included as a detection covariate. The model in bold includes the combination of covariates that we carried through to the multispecies models.

Model name	Covariates						AIC	ΔAIC	*wᵢ* (AIC)
	Range obscured	Ground cover	Termite mound density	Distance to water	Tree cover	Lion activity			
**C19**	**D**	**D**	**D**	**D**		**D**	**2912.17**	**0**	**0.69**
C20	D	D	D	D	D	D	2913.79	1.61	0.31
C16	D	D	D	D	D		2948.09	35.91	<0.001
C10	D	D	D	D			2949.32	37.14	<0.001
C14	D	D		D		D	2954.37	42.19	<0.001
C17	D	D		D	D	D	2955.51	43.33	<0.001
C18	D	D	D		D	D	2974.15	61.98	<0.001
C12	D	D	D			D	2984.01	71.84	<0.001
C7	D	D		D			2989.07	76.9	<0.001
C13	D	D		D	D		2990.99	78.82	<0.001
C11	D	D	D		D		3012.58	100.4	<0.001
C15	D	D			D	D	3015.14	102.96	<0.001
C9	D	D				D	3016.31	104.13	<0.001
C6	D	D	D				3037.11	124.94	<0.001
C8	D	D			D		3055.61	143.44	<0.001
C3	D	D		O			3064.18	152.01	<0.001
C4	D	D			O		3064.95	152.78	<0.001
C5	D	D				O	3064.95	152.78	<0.001
C1	D	D					3065.62	153.45	<0.001
C2	D	D	O				3066.70	154.53	<0.001
C0							3125.82	213.65	<0.001

Abbreviations: AIC, Akaike Information Criterion; wi(AIC), AIC weight.

**TABLE 3:** Honey badger single‐species occupancy model results. Under “Covariates”, ‘O’ means that the variable was included in the model as an occupancy covariate and ‘D’ means it was included as a detection covariate. The model in bold includes the combination of covariates that we carried through to the multispecies models.

Model name	Covariates				AIC	ΔAIC	*wᵢ* (AIC)
	Range obscured	Ground cover	Termite mound density	Lion activity			
HB6	D	D	D	D	816.14	0	0.5
**HB4**	**D**	**D**	**D**		**816.15**	**0.01**	**0.5**
HB5	D	D		D	851.722	35.57	<0.001
HB2	D	D	O		852.90	36.76	<0.001
HB1	D	D			854.58	38.43	<0.001
HB3	D	D		O	856.58	40.43	<0.001
HB0					857.51	41.36	<0.001

Abbreviations: AIC, Akaike Information Criterion; 𝑤𝑖 (AIC), Akaike weight.

**TABLE 4:** Marsh mongoose single‐species occupancy model results. Under “Covariates”, ‘O’ means that the variable was included in the model as an occupancy covariate and ‘D’ means it was included as a detection covariate. The model in bold includes the combination of covariates that we carried through to the multispecies models.

Model name	Covariates				AIC	ΔAIC	*wᵢ* (AIC)
	Range obscured	Ground cover	Distance to water	Lion activity			
**MM4**	**D**	**D**	**D**		**825.60**	**0**	**0.53**
MM6	D	D	D	D	827.32	1.72	0.22
MM1	D	D			828.81	3.21	0.11
MM5	D	D		D	830.12	4.52	0.06
MM3	D	D		O	830.57	4.97	0.04
MM2	D	D	O		830.72	5.12	0.04
MM0					857.51	31.91	<0.001

Abbreviations: AIC, Akaike Information Criterion; 𝑤𝑖 (AIC), Akaike weight.

Once we finalized the covariates for each of the four species, we evaluated the potential for interspecific interactions in multispecies models (Rota et al., 2016; Table 5). We fit these models in R v4.3.0 (R Core Team, 2023) using the occuMulti function in the “unmarked” package (Fiske & Chandler, 2011; Kellner et al., 2023) and compared them based on AIC. The first set of models assumed all four species occur independently of one another: M0 was a completely null model (no occupancy or detection covariates), and M1 included the covariates as determined from the single‐species exploration. Our second set of models (M2‐M8) also included these covariates and allowed for occupancy probabilities to vary in relation to the presence or absence of other mesocarnivore species. We considered the constant probability of a single species pair occupying the same site for each species pair individually (models M2–M7) and all species pairs together (M8; Table 5). We also considered additional models that included lion presence as a covariate affecting the conditional probabilities of a single species pair occupying the same site to explore potential effects of apex carnivores on mesocarnivore spatial partitioning. However, these models failed to produce reliable results, likely due to data deficiency, so we excluded them from consideration and were unable to draw conclusions about the effect of lions on mesocarnivore species interactions.

**TABLE 5:** Description of candidate multispecies occupancy models and results. All models except M0 (null) included the two camera placement detection covariates (range obscured and percent cover) and species‐specific detection covariates: Genet – tree cover, Civet – distance to water, termite mound density, and lion activity, Honey badger – termite mound density, and marsh mongoose – distance to water. In these models, we explored different pairwise species co‐occurrence, indicated in the “Interaction” column.

Model name	Interaction	AIC	ΔAIC	*wᵢ* (AIC)
M6	Civet and marsh mongoose	9709.91	0	0.47
M5	Civet and honey badger	9712.38	2.47	0.14
M1	None	9712.42	2.51	0.13
M2	Genet and civet	9713.95	4.04	0.06
M3	Genet and honey badger	9714.06	4.15	0.06
M4	Genet and marsh mongoose	9714.10	4.19	0.06
M7	Honey badger and marsh mongoose	9714.20	4.29	0.06
M8	All pairwise interactions	9716.38	6.47	0.02
M0	None (also excluded detection covariates)	9928.11	218.2	<0.0001

Abbreviations: AIC, Akaike Information Criterion; 𝑤𝑖 (AIC), Akaike weight.

### 2.4 Temporal analysis

To quantify temporal partitioning among mesocarnivores at GNP, we examined the daily activity patterns of the four focal species from camera trap detections. Though all the species are broadly considered nocturnal (Estes, 2012; Kingdon & Hoffmann, 2013), more fine‐scale differences in diel activity could arise from temporal niche partitioning (Carter et al., 2015; Schuette et al., 2013). We considered detections to be independent if they were at least 30 s apart. We used kernel density estimation to model daily activity patterns of the four focal species, as described in Ridout and Linkie (2009). We converted the time of each detection into radians to account for the circularity of the temporal data. We then scaled the times so that $\frac{\pi }{2}$ corresponded to sunrise and $\frac{3\pi }{2}$ corresponded to sunset. Using the scaled times, we created a smoothed nonparametric kernel density distribution of daily activity for each of the four species based on the observation distribution across a 24‐h period. We used these density distributions to calculate the coefficient of overlap, ${\hat{D}}_{4}$, which ranges from 0 (no temporal overlap between a species pair) to 1 (complete temporal overlap between a species pair). We consider ${\hat{D}}_{4}$ ≥ 0.80 to be high overlap and ${\hat{D}}_{4}$ between 0.50 and 0.79 to be moderate overlap (following Allen et al., 2018). We used 10,000 bootstrapped samples to calculate the 95% confidence intervals. We conducted this analysis in R v3.6.3 (R Core Team, 2020) using the “overlap” package (Ridout & Linkie, 2009).

## 3 RESULTS

Across the four focal species, there were 1984 total independent camera trap records over 6862 trap nights (Table 6). Civets and genets were detected at the majority of the camera traps, while honey badgers and marsh mongoose were detected at approximately half of the camera traps (Table 6).

**TABLE 6:** Traits and detection records for each of the mesocarnivore species documented on camera traps in Gorongosa National Park, Mozambique. Naive occupancy corresponds to the proportion of camera traps at which a species was detected.

Common name	Scientific name	Family	Mass (kg)	Naive occupancy	Detections	Detection rate (per 100 trap‐nights)
African civet	*Civettictis civetta*	Viverridae	12–15^a^	0.88	612	8.92
Large‐spotted genet	*Genetta maculata*	Viverridae	2^b^	0.92	1203	17.53
Honey badger	*Mellivora capensis*	Mustelidae	6–14^c^	0.50	84	1.22
Marsh mongoose	*Atilax paludinosus*	Herpestidae	2.5–4.1^a^	0.55	85	1.24

^a^ Myers et al. (2023).

^b^ Caro and Stoner (2003).

^c^ Begg et al. (2005).

### 3.1 Spatial analysis

**FIGURE 3:**
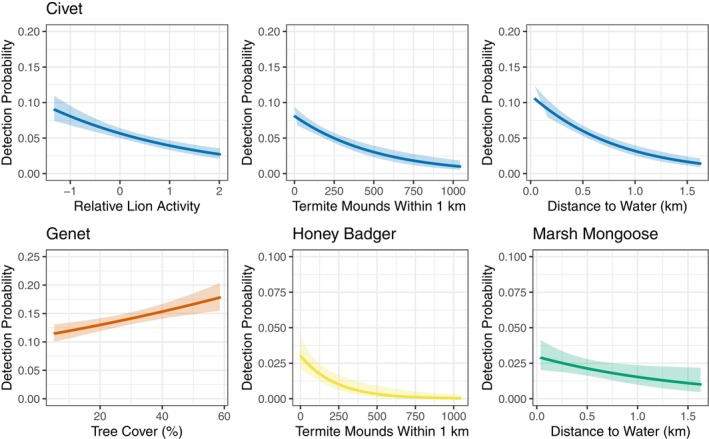
Marginal detection probabilities of each of the four focal mesocarnivore species in Gorongosa National Park, Mozambique, in relation to environmental covariates, based on the top‐ranking species interaction multispecies occupancy model, M6. Solid lines represent the predicted detection probability and lighter ribbons surround 95% confidence intervals. All variables not included in a plot are fixed at their observed mean. We varied the *y*‐axis scale by species for legibility, given differences in detection probabilities across species.

For our single species exploration, including covariates in the occupancy formulas did not improve substantially upon the model that only included the camera placement detection covariates (Models G1, C1, HB1, MM1; Tables 1, 2, 3, 4). However, several detection covariates appeared in the best single‐species models: genet – tree cover, civet – termite mound density, distance to water, and lion activity, honey badger – termite mound density, and marsh mongoose – distance to water (Tables 1, 2, 3, 4). We therefore included these covariates in subsequent multispecies models. Civet detection probability decreased with lion presence, termite mound density, and distance to water; genet detection increased with tree cover (Figure 3). Honey badger detection decreased with termite mound density, while marsh mongoose detection decreased with distance to water (Figure 3).

The null model (M0) without these species‐specific detection covariates ranked ninth of the nine multispecies occupancy models we ran (from low to high AIC) (Table 5). In the best‐performing multispecies occupancy model (with the lowest AIC, M6), the occupancy probabilities of the four focal species were independent of each other, except for civets and marsh mongooses. This model accounted for constant conditional probabilities of these two species occupying the same site (Table 5). However, it is important to note that this interaction term had a large standard error (Table 7). Models M5 and M1 ranked second and third, respectively (Δ AIC = 2.47 and 2.51). Model M5 included an interaction between civets and honey badgers (instead of civets and marsh mongooses). Model M1 included no species interactions.

**TABLE 7:** Detailed results for the best‐performing model (M6), which included species‐specific detection covariates and the co‐occurrence of one species pair (civet and marsh mongoose).

Species	Covariate	Estimate	SE	z	*p*(>|*z*|)
*Occupancy*					
Genet	Intercept	2.41	0.47	5.10	<.0001
Civet	Intercept	1.14	0.56	2.04	<.1
Honey badger	Intercept	0.73	0.42	1.71	<.1
Marsh mongoose	Intercept	−7.36	24.60	−0.30	.77
Civet and marsh mongoose	Intercept	8.19	24.60	0.33	.74
*Detection*					
Genet	Intercept	−1.81	0.04	−41.14	<.0001
	Range obscured	−0.048	0.083	−0.58	.56
	Ground cover	−0.044	0.040	−1.12	.26
	Tree cover	0.14	0.037	3.84	<.001
Civet	Intercept	−2.81	0.068	−41.68	<.0001
	Range obscured	−0.21	0.12	−1.67	<.1
	Ground cover	0.40	0.058	6.91	<.0001
	Termite mound density	−0.44	0.073	−6.055	<.0001
	Distance to water	−0.54	0.069	−7.91	<.0001
	Lion activity	−0.33	0.054	−6.12	<.0001
Honey badger	Intercept	−4.29	0.21	−20.93	<.0001
	Range obscured	−0.33	0.29	−1.12	.26
	Ground cover	−0.34	0.20	−1.73	<.1
	Termite mound density	−0.95	0.25	−3.77	<.001
Marsh mongoose	Intercept	−3.85	0.14	−26.92	<.0001
	Range obscured	−1.19	0.41	−2.86	<.01
	Ground cover	−0.012	0.15	−0.078	.94
	Distance to water	−0.26	0.13	−2.02	<.1

### 3.2 Temporal analysis

**FIGURE 4:**
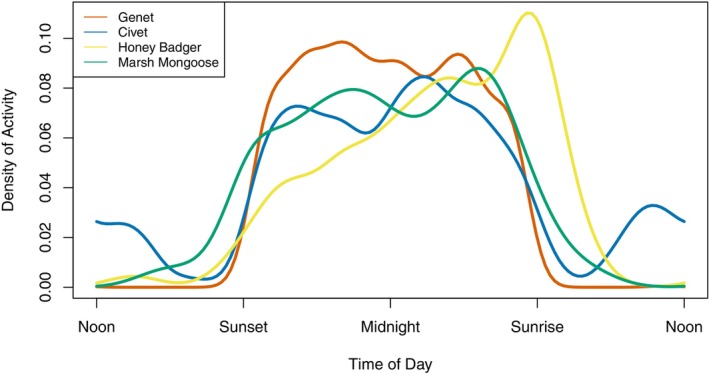
Daily activity patterns for the four focal mesocarnivore species in Gorongosa National Park, Mozambique, across all detections.

Each of the focal mesocarnivores was most active between sunset and sunrise, as expected. The pairwise activity pattern overlap was high for all species pairs (${\hat{D}}_{4}$ ≥ 0.80), except between genets and honey badgers, and between civets and honey badgers, which had moderate overlap (${\hat{D}}_{4}$ = 0.73 [95% CI 0.64–0.83] and 0.75 [95% CI 0.67–0.85], respectively) (Figure 4; Table 8). Civets were the only one of the four species to exhibit any noticeable activity during the day, with a small daytime peak. Honey badgers had a small activity spike around sunrise.

**TABLE 8:** Coefficient of overlap, with 95% confidence interval (CI), for all species pairs considered. Values in bold indicate high overlap in daily activity patterns between the two species.

Species pair	${\hat{D}}_{4}$	Lower CI	Upper CI
Genet and civet	**0.81**	0.79	0.85
Genet and honey badger	0.73	0.64	0.83
Genet and marsh mongoose	**0.86**	0.82	0.97
Civet and honey badger	0.75	0.67	0.85
Civet and marsh mongoose	**0.84**	0.81	0.93
Honey badger and marsh mongoose	**0.80**	0.73	0.96

## 4 DISCUSSION

We found limited evidence of spatial or temporal partitioning among the four most common mesocarnivore species – civet, genet, honey badger, and marsh mongoose – in Gorongosa National Park, Mozambique. In fact, there was some evidence for a positive relationship between civet and marsh mongoose occupancy, and all species pairs exhibited moderate to high temporal overlap. This is perhaps an unsurprising result, given that these species are widespread throughout the study area and are all nocturnal (Davis et al., 2018). In addition, spatiotemporal niche partitioning may only be necessary in ecosystems where species are in competition for limited resources, and especially systems in which apex predators further restrict mesocarnivore resource and space use (Brodie et al., 2018; Schuette et al., 2013). Large carnivore densities remain low in our study area, where wildlife populations are still recovering from recent civil conflict (Bouley et al., 2018, 2021). However, the complexity of African carnivore guilds may limit the strength of intraguild interactions and top‐down effects even in intact systems (Comley et al., 2020b; Welch et al., 2023). Furthermore, dietary and habitat niche differentiation among species may also facilitate coexistence and reduce competition (Brodie et al., 2018; Comley et al., 2020a, 2020b; Owen‐Smith & Mills, 2008).

The observed relationships between species detection probabilities and environmental covariates generally followed our expectations and add to our growing understanding of African mesocarnivore ecology. Civets had overall high occupancy throughout the study site, with higher detection probabilities near water and lower detection probabilities with increasing lion activity. Indeed, civets are reported to be adept swimmers that consume a variety of aquatic prey and are known to be preyed on by lions (Kingdon, 1977; Ray, 2013; Rosevear, 1974). Civets were also the only mesocarnivore species to exhibit a small daytime peak in activity. This diurnal activity may allow them to avoid lions, which are nocturnal hunters (Slotow & Hunter, 2009). Surprisingly, both civets and honey badgers had lower detection probabilities in areas with more termite mounds, despite the potential of termite mounds to serve as an important food resource (Fleming & Loveridge, 2003; Kingdon & Hoffmann, 2013; Okullo et al., 2013). This finding may suggest that there are sufficiently abundant resources in the park that these mesocarnivores do not need to associate with termite mounds. Additionally, the camera traps may not capture the spatiotemporal scales at which animals associate with termite mounds. Genets are the most arboreal species among the four studied here, and they had higher detection probability in areas with more tree cover (Admasu et al., 2004; Maddock & Perrin, 1993). Finally, marsh mongoose have a well‐established preference for habitats with access to water (Baker & Ray, 2013; Kingdon, 1977; Stuart & Stuart, 2003a, 2003b), which explains their higher detection probability near water. In GNP, differences in fine‐scale habitat use may allow mesocarnivore species to coexist in space and time while reducing competition.

We found that civets and marsh mongooses had higher conditional occupancies at sites where the other species was also recorded, contrary to our expectation of spatial partitioning. This co‐occurrence may be related to similar habitat preferences, with both species known to inhabit areas with adequate water supply (Baker & Ray, 2013; Ray, 2013). However, there was a high standard error associated with the co‐occurrence parameter estimate, suggesting that the evidence for this interaction is weak despite it appearing in the best model. We also found that all species had a high degree of temporal overlap, as all were nocturnal. Other factors may affect mesocarnivore diel activity patterns more strongly than competition, including evolutionary constraints and adaptations to nighttime activity, climate conditions, and the activity patterns of both potential prey and larger carnivores (Haswell et al., 2020; Monterroso et al., 2013; Penido et al., 2017). At Gorongosa, such factors seem to have played a larger role in determining mesocarnivore nighttime activity than intraguild temporal partitioning.

Factors other than spatial or temporal partitioning, like specific dietary preferences, foraging strategies, or finer‐scale habitat specialization, may help explain how these mesocarnivore species are able to coexist (Angelici & Luiselli, 2005; Cronk & Pillay, 2019; Estes, 2012). For example, genets are the most arboreal of the group, which may allow for fine‐scale spatial partitioning in the vertical dimension (Maddock & Perrin, 1993). Furthermore, although all of the focal species are carnivores, their diets are varied: genets are primarily carnivorous, with a diet focused on small mammals and insects, while civets are more opportunistically omnivorous (Kingdon & Hoffmann, 2013). Honey badgers are also carnivorous and have the widest range of prey body sizes, from insect larvae to juvenile ungulates (Begg et al., 2003). Finally, marsh mongoose diets include mostly aquatic prey, specifically crustaceans (Angelici, 2000; Stuart & Stuart, 2003a, 2003b). Such coexistence of sympatric carnivore species due to dietary differences has been reported in other systems as well, including in the neotropics (Silva‐Pereira et al., 2011). Additionally, species may undergo ecological character displacement and adopt different dietary patterns in the presence of other species to enable coexistence, given sufficient resources (Pfennig et al., 2006). Further work investigating the potential for dietary partitioning among mesocarnivores in GNP is needed to understand its role in supporting their coexistence.

Abundant resources in an ecosystem may lead to fewer competitive interactions among mesocarnivores and less resource partitioning (Brodie et al., 2018). The results of our study, with limited evidence for effects of one mesocarnivore species on another, suggest that there may be abundant resources for mesocarnivores in GNP, perhaps due to low densities of large carnivores post‐conflict. In Botswana, Rich et al. (2017) found that resource availability had a greater effect on carnivore occupancy than the presence of competitor species. In that system, civet occupancy was positively related to similarly sized species' detection rates only during the wet season but not the dry season, which can potentially be explained as a result of lower resource availability and therefore greater competition in the dry season (Rich et al., 2017). Indeed, in a system with abundant resources, Monterroso et al. (2020) found that spatial coexistence was detected over five times more frequently than competitive avoidance among mesocarnivore species. With available resources, potentially competing species may modify their diets in the presence of competitor species to reduce niche overlap and competition (Schoener, 1974).

High occupancy probabilities for the four focal species indicate that they are all widespread in the study area, so it is unsurprising that we only found evidence of one species' occupancy affecting the occupancy of another for a single species pair. It is also possible that our sample sizes of camera sites and mesocarnivore detections were insufficient to detect subtle, fine‐scale patterns of spatiotemporal partitioning. Recent camera trap modeling advances allow for simultaneous analysis of both space and time and may reveal nuanced interactions among species like African mesocarnivores (Kellner et al., 2022; Parsons et al., 2022). However, many of these complex models have extensive data requirements beyond the scope of the current study. Additional data collection in Gorongosa and in other study sites across Africa may provide further insight into the nature of mesocarnivore competition and coexistence.

Furthermore, this study is limited to a single dry season to better meet assumptions of closure (Sollmann, 2018), but patterns of spatiotemporal partitioning in mesocarnivores may vary seasonally (Rich et al., 2017). This potential seasonal variation may help explain the difference between our work and that of Raimundo (2020), which found spatial avoidance between civets and genets in GNP earlier in the year (June–October). During the wet season at GNP, approximately 20% of the park experiences seasonal flooding (Stalmans & Beilfuss, 2008), which may force all mesocarnivore species into closer proximity and increase the likelihood of interaction effects. Further work should include multiple seasons to assess potential differences associated with intra‐annual variation.

Our study design and sample sizes (in terms of cameras and lion detections) limited our ability to reliably evaluate the effect of lion activity on interactions among the mesocarnivore guild. However, we found that lion activity had a negative effect on civet detection probability. Civets were ubiquitous across the study site, sharing space with lions, and the observed effect of lions on detection probability is likely the result of lower civet density or reduced civet movement in areas with more lion activity. The presence of a larger, dominant predator species often restricts the distribution and alters the behavior of smaller predators (Sergio & Hiraldo, 2008). Mesocarnivore suppression has been reported in several studies (e.g., Dröge et al., 2017; Vanak et al., 2013) with smaller predators spending less time in resource‐rich areas to evade larger predators. Other studies have reported both positive and negative effects of large carnivores on mesocarnivore activity, with mesocarnivore behavioral flexibility in response to large carnivore density (Comley et al., 2020b; Welch et al., 2023). The lack of evidence for an effect of lions on the other mesocarnivores at GNP is perhaps not surprising due to the very low density of larger predators, with only lions remaining from the pre‐conflict large carnivore guild (Bouley et al., 2018). Additionally, we were only able to use a coarse proxy for relative lion activity that did not align temporally with the camera trap data, which likely limited our ability to detect any effects of lions on mesocarnivore space use. Continued exploration of the relationship between apex predator presence and mesocarnivore response in GNP and other systems with different large carnivore guilds will help parse out the role of apex predators in mediating interactions between mesocarnivores.

As the large carnivore guild grows in GNP, both in terms of population densities and species diversity, trophic interactions will likely become more complex and mesocarnivore responses to increased large carnivore presence will likely vary by species (Phillips & Pringle, 2023). Since this study, two packs of African wild dogs were translocated to the park and have rapidly reproduced and split into new packs (Bouley et al., 2021; Stalmans et al., 2019). There is a current population of approximately 250 individuals across several packs, along with approximately 6 leopards, 12 spotted hyenas, and 8 side‐striped jackals. Some mesocarnivore species may have a negative response to these reintroduced large carnivores. Banded mongooses (*Mungos mungo*), for example, run for cover in the presence of larger carnivores like lions, while other species are known to be preyed on by spotted hyenas, including genets and civets (Kingdon & Hoffmann, 2013). Direct competition for resources may be limited between these apex carnivores and the mesocarnivore species in the park due to differences in preferred prey size (Hayward et al., 2006), but they might negatively impact the smaller carnivores' activity by instilling fear (Gaynor et al., 2019) or through intraguild predation (Holt & Polis, 1997; Hunter & Caro, 2008; Palomares & Caro, 1999; Polis, 1981). Such avoidance of larger carnivores may be a reactive, rather than predictive, response to risk (Broekhuis et al., 2013; López‐Bao et al., 2016), which is difficult to capture using traditional camera trap methods that do not provide sufficiently fine‐scale information.

Large carnivores may also facilitate mesocarnivores through carrion provisioning (Fàbregas et al., 2017; Prugh & Sivy, 2020; van Dijk et al., 2008). In GNP, we have observed several mongoose species, civets, servals and side‐striped jackals feeding on carrion. However, additional carrion in the system may also lead to increased potential for interference competition between larger scavenger species, like spotted hyenas and jackals, and the mesocarnivore scavenger species. Whether large carnivores suppress or facilitate mesocarnivores may be dependent on site‐specific factors: Welch et al. (2023) found that apex predators both positively and negatively influenced the detection of black‐backed jackals (*Canis mesomelas*) at sites with different densities of large carnivores. We may therefore see either increased spatial or temporal overlap among mesocarnivores as they avoid the larger carnivores (Swanson et al., 2014; Vanak et al., 2013), or reduced competition as a result of higher resource availability (Yarnell et al., 2013). Increased overlap may force more reactive risk responses from subordinate species at finer scales (i.e., at carcass sites) than can be captured by camera trap data to avoid confrontation with more dominant species (Broekhuis et al., 2013; López‐Bao et al., 2016).

By quantifying spatial and temporal patterns of the mesocarnivore guild in Gorongosa National Park, this work sheds light on the dynamics of an ecosystem recovering from decades of disturbance (Stalmans et al., 2019). Given the potential for global release of mesocarnivores as a result of large carnivore declines, such studies are essential to providing a more detailed understanding of mesocarnivore ecology (Prugh et al., 2009; Prugh & Sivy, 2020). GNP presented a unique opportunity to study an ecosystem with almost no apex predators – a scenario that is unfortunately becoming more common globally (Gálvez et al., 2017; Prugh et al., 2009). Some have argued that apex predator reintroduction is the only way to restore ecosystems to their former states (e.g., Ritchie et al., 2012) and indeed, research conducted at GNP suggests that the return of apex predators will help restore the communities that existed before conflict‐induced defaunation (Atkins et al., 2019). However, anthropogenic impacts may alter the population size and behavior of large carnivores, which can diminish the strength of the ecological effects of these apex predators in human‐dominated landscapes (Kuijper et al., 2016). Human activity has greatly reshaped the greater Gorongosa ecosystem over time, through hunting, agriculture, climate change, and civil conflict (Daskin & Pringle, 2018; Easter et al., 2019; Gaynor et al., 2021). Although the park is now a protected area, there is still a substantial human footprint in the buffer zone surrounding the park and a smaller footprint in the park from tourism, research, and poaching. Continued monitoring of Gorongosa's mesocarnivore guild to assess its response to ongoing predator reintroductions can shed light on carnivore ecology and inform future conservation efforts. Beyond Gorongosa, further research on mesocarnivore ecology and species interactions is essential for the global conservation of this understudied taxa, particularly as the role of mesocarnivores is shifting amidst both large carnivore declines and reintroductions.

## AUTHOR CONTRIBUTIONS

**Kathryn L. Grabowski:** Conceptualization (equal); data curation (supporting); formal analysis (lead); investigation (lead); methodology (lead); project administration (equal); software (lead); visualization (lead); writing – original draft (lead); writing – review and editing (lead). **Erin M. Phillips:** Conceptualization (supporting); writing – review and editing (supporting). **Kaitlyn M. Gaynor:** Conceptualization (equal); data curation (lead); funding acquisition (lead); investigation (equal); methodology (supporting); software (supporting); supervision (lead); visualization (supporting); writing – original draft (supporting); writing – review and editing (supporting).

## CONFLICT OF INTEREST STATEMENT

The authors have no competing interests to declare.

## Data Availability

All data and code are publicly available on Dryad: https://datadryad.org/stash/share/tuhUCDivJs5lQUA_NjhxSiFnqZOLh9TMZ7E7XmDG34o.
